# Modern Thought in Medicine—Pres’t Powell’s Charge to the Graduates in Medicine

**Published:** 1888-04

**Authors:** 


### MODERN THOUGHT IN MEDICINE.

### PRESIDENT POWELL'S CHARGE TO THE GRADUATES IN MEDICINE

*Gentlemen of the Graduating Class :*

It was a source of great pleasure to me to say to you in connection with the delivery of your Diploma by the Dean, that by authority vested in me by the laws of Georgia, you are now Doctors of Medicine and legally entitled to all the rights and privileges of the profession

Your Alma Mater will not be satisfied with merely seeing you start out fairly and honorab y upon the difficult and far-stretching path that every doctor of medicine must tread before he can make a noble and enviable success of his profession.

We would have you ever remember, that the true physician is not only a man of knowledge, but is also one who continually aspires after more knowledge.

He is a man of "noble discontent" because he is not satisfied with his attainments and feels that there are yet greater heights in medical lore, and its cognate sciences, which he has never reached.

To assend this eminence his efforts will be directed as long as life lasts, or until he has toiled up to the grand summit, and planting his feet upon its apex, lifts his head and cries in triumphant tones, "Eureka!"

Yet our profession has often been charged by the public with slowness in seizing upon advanced thoughts, and utilizing constantly occurring expediences in progressive medicine.

It is said to be too stubbornly wedded to fossil ideas, methods and remedies of the past.

The profession is greatly to blame for this misconception and adverse criticism on the part of the public, inasmuch as it has no specific methods of keeping the people informed upon the great advance that has been made in medicine in the last twenty years. In the "grand sweep of modern thought," medical science is not a laggard in the procession, but is marching boldly and steadily to the front under the inspiration of nobler endeavors and higher attainments.

It is a mistake to call medicine in toto an inexact science as some have done; for while there can be such a thing as an incomplete art, a science, per-se, is never inexact. A science is composed of facts, and it has grasped these truths and made them its own by patient, intelligent experiment and unmistakable demonstration. Then it truly becomes medical science, going on from the known to find and prove the unknown. So medicine is not inexact in all its branches.

In the great complexity of disease, with its boundless field for exploration, the medical scientist has discovered much ground upon which he can now safely stand, and this leads me, young gentlemen, to speak briefly of "Thought in Modern Medicine." The cause and effect of disease is the most important study in the profession, and it is believed that this is now more prominently and intelligently studied and discussed than ever before in the history of medicine.

The profession is wider and more discriminate in all pathological departments and in the understanding of the all pervading laws of nature in the presence and the cure of disease. To more fully investigate these natural causes, is the important but difficult undertaking to which modern medical science is directing its labors with an energy, zeal and intelligence unknown in its past endeavors. When we see the *expose* of what seems to be a marvelous exhibition of legerdemain, we are astonished to find how simple is the secret, and though the unfolding of the complex mysteries of nature in the cause and effect of disease is so vast and difficult a work, when we do have one of the secrets revealed to us, we are almost startled at its wonderful simplicity.

Equally striking are the bountiful and beneficent means the "Great Silent Mother" gives to the skillful physician and the intelligent patient, so that they may co-operate with her in the removal or prevention of disease ; and herein also is a simplicity as beautiful as it is benign.

Thus, while all intelligent physicians know that nature requires a sick person to be kept hopeful, cheerful, quiet, with plenty of undisturbed sleep, sufficient nourishment of a kind to sustain nutrition, a little physic intelligently given, if any should be needed at all ; they also know that from want of information on the part of the patient's nurses, and sometimes of the medical quack, scores of sick people are kept awake, and continually excited, are fussed and worried over, dosed and talked into untimely graves.

Simple hygienic agencies may appear to the quack doctor of little or no moment, but the physician knows that they are based upon truly scientific principles, and that their observance is of as much therapeutical importance as the employment of drugs.

The natural history of disease is distined to become a prominent feature in the character of progressive medicine ; and the profession will agree, that to master this branch of medical science, it must learn profoundly of Nature, it must employ her laws, if it would become victor over man's natural foe—disease.

As Dr. Rush says, disease is a lawless enemy. It is, in fact, a defiant and malignant rebel against all of Nature's most gracious and beautiful statutes, and it is ever on the alert to fall upon and destroy the mental and physical health of man.

Instead of violent spasmodic assaults, or rushing helter skelter upon this powerful and ever vigilant foe, thinking to put him to flight with an indiscriminate shower of drugs, the intelligence of modern medicine instructs the physician in a better system of medical tactics. It teaches him that to meet the aggressions of the enemy, he must bravely and deliberately bring against them forces of superior power and character, and that these forces must be gathered from the kingdom of Nature, of which she is sole earthly sovereign.

The skillful physician is her Major General, commanding whatever beneficent remedial agents that may be found in her realm; makes his strategetic movements in pursuance of her orders, and obeys the slightest intimation of her will. In fact, upon accomplished strategy, as taught by this sovereign queen, the learned physician greatly relies, holding the ranks of the pharmacopeia as a reserve corps to be brought forward and actively employed when they are really needed to aid him in becoming sole master of the field.

The physician, in thus co-operating with Nature, becomes an actual co-worker with the great God of the universe.

It is a sublime position for man's aspiration, young gentlemen, and to attain it is worth many years of patient toil and profound study. It is a grand work, and because of its divine benevolence and exalted character, it should be undertaken in a reverent spirit, with a pure heart and a noble ambition.

The doctor is not only a commanding officer as herein stated, but, as the name implies, he is a teacher as well as a minister of physic.

This makes three golden links complete—nature taught by God, the doctor taught by Nature, and the people instructed by the doctor.

In modern medicine, it is gratifying to see that more importance is being attached to the intelligent and thorough study of medical chemistry. However proficient you may be in other branches of medical lore, it is necessary for you to know such apparently simple truths as that the basic acid in the apple and some other fruits is greatly beneficial in certain disorders, and in keeping the system in good health, and that one part of a chicken as food is of more benefit to one patient, and another part to some patient afflicted with a different disease, and scores of similar discriminations that might be mentioned in the chemical philosophy of remedies, without which, as some of the profession have pertinently said, the practice of medicine would be "a mere gamble for human life."

Modern thought in medicine is refuting the old idea that disease is the consequence of life. Sanitary science now boldly asserts that man is born to health and longevity—that disease is abnormal, and death, except from old age, is accidental, and that both are preventable by human agencies.

As men and women intelligently learn that humanity is responsible for much of its own sickness and premature death, it will not seem to be extreme, as it has been said by one, that the time is coming when individuals and communities will consider it a disgrace to be sick.

The lamented Dr. Samuel Gross, the father of American Surgery, said to the young men of America, that the great question of the day was not this or that operation which had reflected so much glory on American medicine, but that it was preventive medicine, the hygiene of our persons, our families, our dwellings and our streets, in a word, our surroundings, whatever or wherever they may be, in city, town, hamlet or country, and the people must be roused upon this subject.

It is the intelligent and humane physician, whether young or old, who must awaken the people to this fact, and teach them that the physical, moral and mental development of individuals, families, communities, and even the nation itself, is involved in this great and momentous question.

It has been well said in high medical circles, that the services of the noble and unselfish physician as a Sanitarian, will be in still increasing demand for generations to come, and the sooner physicians adjust their ministrations and fees to that demand, the better it will be for themselves and their patrons, and the prospects of humanity will perceptibly brighten.

I wish I had time to enlarge upon this idea. It is an exalting spectacle to mentally contemplate the great God of the universe, who never desires to destroy, but always to save, sitting upon His imperial throne, and with His own gracious omnipotent hand reaching down from the skies, unrolls before us the map of Nature's world-wide kingdom, and invites sanitary science to bravely search for, and reveal its hidden mysteries—to find there the true elixir of life, and holding it to the lips of the people, bid them drink and live to more than threescore years and ten in health and happiness, and vigor of both body and mind.

To be fitted for such a profession as yours is destined to be, your efforts should be made, and your character molded to this end. Never think you know enough in your profession.

Some writer has said that "ambition is always humble when it is about to climb." The more a man knows about his business, the more he will see there is to learn, and if he is worthy of this honorable vocation he will be ever aspiring after the loftiest possible altitude. As you toil and strive for this, hold fast to integrity of princip e and purpose, be brave and hopeful, and have confidence in your noble aims and own capacity. A man must have faith in both his possibility and responsibility. Have a high estimate of personal character in yourselves and others.. Just and cordial appreciation of every noble man you know, will add a lustre to your own nobility of soul. No man is ever better than he deserves to be—you will therefore choose your associates and your exemplar from just such men as you wish to copy in your own life and character. Do not make the mistake of supposing your course and character are right because you may be commended by men whom you do not, and cannot wholly respect and honor. It is better to have the frowns of an enemy than the approval of this kind of a friend. While you may respect the position itself a man has gained by objectionable methods, you cannot in conscience honor him who holds it unworthily.

King Solomon's death-bed injunction to his princely son David comprises the whole—"Be a man." Grasp that one word in all its wide and exalted significance. Make it the polar star of your ambition, and under God, you will find its fruition, and bless the world as you will be blessed by heaven.

My young brethren I bid you God speed in your noble mission of ministering to the suffering ones of earth. Be true to yourselves and your profession—be true to your own and your father's and mother's God, and may Heaven's richest blessings be yours through life.

Its sad to bid you good-bye, but my heart-felt interest will follow wherever you go, and I pray you to live so that when this earthly life is over with us all, we will greet each other in that "beautiful land" where farewells are never spoken, and we will find life indeed for evermore.

